# Sex-Specific Associations Between Inter-Individual Differences in Heart Rate Variability and Inter-Individual Differences in Emotion Regulation

**DOI:** 10.3389/fnins.2018.01040

**Published:** 2019-01-22

**Authors:** Alexander Lischke, Matthias Weippert, Anett Mau-Moeller, Stefanie Päschke, Robert Jacksteit, Alfons O. Hamm, Rike Pahnke

**Affiliations:** ^1^Department of Psychology, University of Greifswald, Greifswald, Germany; ^2^Department of Sport Science, University of Rostock, Rostock, Germany; ^3^Department of Orthopaedics, University Medicine Rostock, Rostock, Germany

**Keywords:** emotion regulation, suppression, reappraisal, vagus nerve, heart rate variability

## Abstract

Neurobiological theories suggest that inter-individual differences in vagally mediated heart rate variability (vmHRV) have the potential to serve as a biomarker for inter-individual differences in emotion regulation that are due to inter-individual differences regarding the engagement of prefrontal and (para-)limbic brain regions during emotion processing. To test these theories, we investigated whether inter-individual differences in vmHRV would be associated with inter-individual differences in emotion regulation. We determined resting state vmHRV in a sample of 176 individuals that had also completed a short self-report measure of reappraisal and suppression use. Resting state vmHRV was derived from short-term (300 s) and ultra-short-term (120 s, 60 s) recordings of participants’ heart rate to determine the robustness of possible findings. Irrespective of recording length, we found that an increase in resting state vmHRV was associated with an increase in self-reported reappraisal but not suppression use. However, this association was only evident among male but not female participants, indicating a sex-specific association between inter-individual differences in resting state vmHRV and inter-individual differences in self-reported emotion regulation. These findings, which are consistent with previous ones, support theoretical claims that inter-individual differences in vmHRV serve as a biomarker for inter-individual differences in emotion regulation. Combing (ultra-)short-term measures of resting state vmHRV with short self-report measures of emotion regulation may, thus, be useful for researchers who have to investigate the neurobiological mechanisms of emotion regulation in a time- and resource-efficient manner.

## Introduction

As social beings, we interact on a daily basis with other individuals. Although these interactions are often rewarding (e.g., interacting with a caring partner), they may also turn out to be frustrating (e.g., interacting with a stubborn child). Whereas in some situations it may be appropriate to show our frustration (e.g., in an argument with our partner), in other situations it may be more appropriate to control our emotions that are fueled by our frustration (e.g., in an argument with our child). The ability to regulate our emotions, thus, determines the course of our social interactions (Gross, 2002), which may have positive outcomes after successful emotion regulation and negative outcomes after unsuccessful emotion regulation (Butler et al., 2003; Gross and John, 2003; English et al., 2012). Unsuccessful emotion regulation may also result in clinical and subclinical levels of stress, anxiety and depression (Garnefski et al., 2001; Gross and John, 2003; Martin and Dahlen, 2005; Moore et al., 2008; Hofmann et al., 2009; Aldao and Nolen-Hoeksema, 2010), thereby increasing the risk to develop mental disorders in the aftermath of emotionally arousing interactions (Gross, 2002). However, whether we succeed or fail to regulate our emotions crucially depends on the type of strategy we employ for this type of purpose (Gross, 2002). Reappraisal is an emotion regulation strategy aimed at changing the meaning of an emotional event (antecedent-focused strategy), whereas suppression is an emotion regulation strategy aimed at changing the reaction to an emotional event (response-focused strategy). Reappraisal use appears to be more efficient in attenuating emotional distress and physiological arousal than suppression use (Gross, 1998; Butler et al., 2003; Gross and John, 2003; Moore et al., 2008; Hofmann et al., 2009; Aldao and Nolen-Hoeksema, 2010), presumably because reappraisal use is more associated with an increase in prefrontal activity and a decrease in (para-)limbic activity than suppression use (Goldin et al., 2008; Drabant et al., 2009; McRae et al., 2010; Vanderhasselt et al., 2013; Nelson et al., 2015) As a consequence, suppression use is more likely to cause symptoms and disorders of stress, anxiety and depression than reappraisal use (Gross, 2002).

Considering the importance of reappraisal and suppression use for mental health (Aldao et al., 2010; Sheppes et al., 2015), much research has been devoted to identify biomarkers that indicate deficits in emotion regulation. Although abnormal activity changes in prefrontal and (para-)limbic brain regions have been considered as biomarkers for deficits in emotion regulation (Ochsner et al., 2012; Frank et al., 2014), the recording of changes in brain activity require neuroimaging protocols that are difficult to realize without the necessary staff and equipment. Other techniques than neuroimaging ones may be more useful to identify biomarkers for emotion regulation. Electrocardiologic techniques that allow an indirect assessment of changes in brain activity via changes in cardiac activity may represent a promising alternative to neuroimaging techniques because these techniques do not require dedicated staff or equipment (Beauchaine, 2015). Whereas changes in cardiac activity can be assessed within a couple of minutes with mobile or stationary devices that do not require extensive training, changes in brain activity can only be assessed over longer time periods with stationary devices that require extensive training. Although changes in cardiac activity can be measured in several ways, measures related to parasympathic induced changes in heart rate (HR) that are mediated by the vagus nerve, which are commonly described as vagally-mediated heart rate variability (vmHRV; Shaffer and Ginsberg, 2017), are of particular relevance. Inter-individual differences in vmHRV are associated with inter-individual differences in prefrontal and (para-)limbic brain activity that are implicated in self-regulatory processes (Thayer and Lane, 2009; Thayer et al., 2012; Smith et al., 2017), indicating that inter-individual differences vmHRV may serve as a biomarker for inter-individual differences in emotion regulation (Appelhans and Luecken, 2006; Beauchaine, 2015; Holzman and Bridgett, 2017). It has already been shown that individuals with high vmHRV have fewer difficulties in emotion regulation than individuals with low HRV (Williams et al., 2015), which may explain why individuals with high vmHRV report less stress, anxiety and depression than individuals with low vmHRV (Fabes and Eisenberg, 1997; Oveis et al., 2009; Kok and Fredrickson, 2010; Kogan et al., 2013; Lischke et al., 2018a). It remains, however, unclear whether these differences in stress, anxiety and depression are due to differences in reappraisal and suppression use. It may be possible that individuals with high vmHRV experience less stress, anxiety and depression than individuals with low vmHRV because individuals with high vmHRV use more reappraisal and less suppression for emotion regulation than individuals with low vmHRV. To address this issue, we investigated whether inter-individual differences in vmHRV would be associated with inter-individual differences in reappraisal and suppression use in a large and homogenous sample of participants. Inter-individual differences in reappraisal and suppression use were determined on basis of participants’ self-reports. Inter-individual differences in vmHRV were determined on basis of short-term and ultra-short-term recordings of participants’ resting state HR. This allowed us to test whether associations between inter-individual differences in resting state vmHRV and inter-individual differences in self-reported reappraisal or suppression use would be invariant across recording conditions, thereby indicating the robustness and stability of these associations. We expected that inter-individual differences in resting state vmHRV would be positively associated with inter-individual differences in self-reported reappraisal use and that inter-individual differences in resting state vmHRV would be, if at all, negatively associated with inter-individual differences in self-reported suppression use. We also explored whether participants’ sex would moderate these associations because inter-individual differences in emotion regulation and vmHRV seem to be sex-dependent (Nolen-Hoeksema, 2012; Koenig and Thayer, 2016).

## Materials and Methods

### Participants

As a previous study revealed medium sized associations between inter-individual differences in resting state vmHRV and inter-individual differences in self-reports regarding the *inability* to regulate emotions (Williams et al., 2015), we expected to find similar sized associations between inter-individual differences in resting state vmHRV and inter-individual differences in self-reports regarding the *ability* to regulate emotions. A power analysis with G^∗^Power 3 indicated that we had to recruit 82–92 participants to have sufficient power (1-β = 0.80, α = 0.05) to detect medium sized associations (*r* = 0.030, *f*^2^ = 0.015) in our correlation and regression based analyses (Faul et al., 2007). We only considered Caucasians with an age range of 18–35 years for recruitment, which resulted in the recruitment of 176 participants, 91 male and 85 females (see Table 1). All participants provided written-informed consent to the study protocol that was approved by the ethics committee of the University of Rostock and carried out in accordance with the Declaration of Helsinki.

**Table 1:** Participant characteristics.

	Female participants		Male participants	
	(*n* = 85)		(*n* = 91)	
		
	*M (SE M)*	95% CI	*M (SE M)*	95% CI
Age	23.85 (0.42)	[23.02, 24.68]	25.81 (0.41)	[25.01, 26.62]
BMI	21.49 (0.27)	[20.96, 22.02]	23.90 (0.26)	[23.39, 24.42]
BSI-18	0.50 (0.04)	[0.42, 0.57]	0.35 (0.04)	[0.28, 0.42]
ASQ-REA	3.39 (0.07)	[3.26, 3.53]	3.80 (0.06)	[3.67, 3.93]
ASQ-SUP	2.85 (0.07)	[2.70, 2.99]	3.00 (0.07)	[2.86, 3.13]
RMSSD-300	49.56 (3.42)	[42.76, 56.35]	43.37 (2.43)	[38.53. 48.21]
RMSSD-120^a^	54.22 (4.00)	[46.27, 62.17]	45.92 (2.76)	[40.43. 51.42]
RMSSD-060^a^	55.63 (4.09)	[47.49, 63.77]	46.85 (2.72)	[41.44. 52.26]
Log-RMSSD-300	1.62 (0.03)	[1.56, 1.67]	1.57 (0.03)	[1.52, 1.63]
Log-RMSSD-120^a^	1.65 (0.03)	[1.59, 1.71]	1.60 (0.03)	[1.54, 1.65]
Log-RMSSD-060^a^	1.66 (0.03)	[1.61, 1.72]	1.61 (0.03)	[1.55, 1.66]


*ASQ-REA, Affective Style Questionnaire – Reappraisal Scale (Hofmann and Kashdan, 2010; Graser et al., 2012); ASQ-SUP, Affective Style Questionnaire – Suppression Scale (Hofmann and Kashdan, 2010; Graser et al., 2012); BMI, body mass index; BSI-18, Brief Symptom Inventory 18 (Franke et al., 2017); 95% CI, 95% confidence interval; RMSSD-300, root mean square of successive differences between consecutive heart beats that had been derived from 300 s lasting heartrate recordings; RMSSD-120, root mean square of successive differences between consecutive heart beats that had been derived from 120 s lasting heartrate recordings; RMSSD-060, root mean square of successive differences between consecutive heart beats that had been derived from 60 s lasting heartrate recordings; Log-RMSSD-300, log-transformed root mean square of successive differences between consecutive heart beats that had been derived from 300 s lasting heartrate recordings; Log-RMSSD-120, log-transformed root mean square of successive differences between consecutive heart beats that had been derived from 120 s lasting heartrate recordings; Log-RMSSD-060, log-transformed root mean square of successive differences between consecutive heart beats that had been derived from 60 s lasting heartrate recordings. ^*a*^Data was missing for one male participant due to technical difficulties.*

### Procedure

Participants were instructed to abstain from alcohol and drugs in the 24 h preceding the testing day. On the testing day, participants were asked to use the bathroom to control for the effects of bladder filling and gastric digestion on resting state vmHRV (Quintana and Heathers, 2014). Thereafter, participants were seated in a comfortable chair and prepared for a HR recording that was used to determine participants’ resting state vmHRV. As recently recommended (Quintana et al., 2016), participants were instructed to breathe spontaneously and to keep their eyes open during the HR recording. Thereafter, participants completed self-report measures of psychopathology (Brief Symptom Inventory 18, BSI-18; Franke et al., 2017) and emotion regulation (Affective Style Questionnaire, ASQ; Hofmann and Kashdan, 2010; Graser et al., 2012).

### Heart Rate Variability

Using a portable HR monitor (RS 800, Polar Electro Oy, Kempele, Finland), participants’ HR was recorded for 300 s at a sampling rate of 1000 HR. Portable HR monitors are as accurate in HR recording as stationary HR monitors (Weippert et al., 2010; Quintana et al., 2012), indicating a valid and reliable recording of the HR data. Device-specific software (Polar ProTrainer 5 Polar Electro Oy, Kempele, Finland) was used to transfer the recorded data to a computer for further data processing with Kubios HRV 2.2 (Tarvainen et al., 2014). Following established guidelines (Task Force of the European Society of Cardiology, 1996), the recorded data was artifact corrected and subjected to a time-domain analysis for the determination of the root mean square of successive differences between consecutive heart beats (RMSSD). RMSSD was determined on basis of different recording lengths, including a short-term recording that lasted 300 s (RMSSD-300) and two ultra-short-term recordings that lasted 120 s (RMSSD-120) and 60 s (RMSSD-060). Although multiple HRV measures can be derived from resting state HR recordings (Shaffer and Ginsberg, 2017), no other HRV measures than RMSSD were considered for statistical analysis to avoid interpretational issues from the use of multiple (inter-correlated) HRV measures. Compared to other HRV measures, RMSSD is the most robust measure of parasympathetic induced changes in HR that are mediated by the vagus nerve (Penttila et al., 2001; Bertsch et al., 2012). RMSSD is also one of the few HRV measures that can be derived from short-term *as well as* ultra-short-term resting state HR recordings in a valid and reliable manner (Bertsch et al., 2012; Munoz et al., 2015). RMSSD was, thus, as recommended (Task Force of the European Society of Cardiology, 1996), the vmHRV measure of interest.

### Psychopathology

Participants’ psychopathology was assessed with the BSI-18 (Franke et al., 2017), an 18 item comprising self-report measure of current psychopathological distress [BSI-18: α = 0.84]. Each item asked for the severity of anxious, depressive and somatoform symptoms within the last 7 days (e.g., “*Within the last 7 days, how much did you suffer from feelings of loneliness?*”). Participants had to indicate how much they were suffering from these symptoms by using a 5-point Likert that ranged from 0 (“*not at all*”) to 4 (“*extremely*”). Ratings of these symptoms have been shown to be associated with formal diagnoses of mental disorders (Prinz et al., 2013), implying a valid and reliable assessment of participants’ psychopathological distress.

### Emotion Regulation

Participants’ emotion regulation abilities were assessed with the ASQ (Hofmann and Kashdan, 2010; Graser et al., 2012), a self-report measure that differentiates between various emotion regulation strategies. Reappraisal strategies aimed at changing the meaning of an emotional event were assessed with a scale that comprised 5 items [ASQ-REA: α = 0.77], whereas suppression strategies aimed at changing the reaction to an emotional event were assessed with a scale that comprised 9 items [ASQ-SUP: α = 0.83]. The items described these emotion regulation strategies in terms of statements (e.g., “*I can avoid getting upset by taking a different perspective on things*” or “*I often suppress my emotional reactions to things*”). Participants had to indicate how much they agreed with each statement by using a 5-point Likert scale that ranged from 0 (“*not true for me at all*”) to 4 (“*extremely true of me*”). Agreement with these statements has been shown to be associated with the actual use of the respective emotion regulation strategies (Szasz et al., 2011, 2012), indicating a valid and reliable assessment of participants’ reappraisal and suppression use.

### Statistical Analysis

SPSS 22 (SPSS Inc., Chicago, IL, United States) was used for all analyses. Analyses of variances (ANOVAs) were run to investigate inter-individual differences in age, body mass index, psychopathology, self-reported emotion regulation abilities and resting state vmHRV. Correlation and regression analyses with bootstrapping (1000 samples) were run to analyze associations between inter-individual differences in resting state vmHRV and inter-individual differences in self-reported reappraisal or suppression use. Precautions were taken to control for inter-individual differences in age, body mass index and psychopathology that may have affected the results of the correlation and regression analyses (Licht et al., 2008, 2009; Abhishekh et al., 2013; Koenig et al., 2014). Whereas correlation analyses were used to explore these associations in male and female participants, regression analyses were used to test whether these associations differed between male and female participants. In the respective regression models, inter-individual differences in age, sex, body mass index, psychopathology and resting state vmHRV were used as predictors and self-reported reappraisal or suppression use as criterion. All predictors, with the exception of sex, were z-transformed to control for multicollinarity (Aiken and West, 1991). The first block of predictors comprised sex, age, body mass index, psychopathology and resting state vmHRV, whereas the second block of predictors comprised the interaction of sex and resting state vmHRV (Aiken and West, 1991). Significant interactions between the predictor sex and the predictor resting state vmHRV were investigated with simple slope analyses (Aiken and West, 1991). To determine the robustness of possible associations between inter-individual differences in resting state vmHRV and inter-individual differences in self-reported reappraisal or suppression use, the correlation and regression analyses were run with short-term (ST) and ultra-short-term (UT) measures of resting state vmHRV (vmHRV-ST: RMSSD-300; vmHRV-UT: RMSSD-120, RMSSD-060). Correspondence between the different resting state vmHRV measures was assessed on basis of correlation analyses that comprised bivariate correlations and intra-class correlations (ICC: absolute agreement, two-way ANOVA; Fleiss et al., 2013). To account for deviations from normality distribution, the resting state vmHRV measures were log transformed (log 10) before all analyses. The significance level for the analyses was set at *p* ≤ 0.05. In addition to the significance value *p*, 95% confidence intervals (CI) and effect size measures (*r*, *R*^2^, Δ*R*^2^, *B*,$\eta_{p}^{2}$) were determined to facilitate the interpretation of (marginally) significant findings (Cohen, 1988; Cumming, 2013).

## Results

### Participant Characteristics

A series of one-way ANOAVs was run to investigate inter-individual differences in participant characteristics (see Table 1). Male participants were slightly older than female participants [*F*(1,174) = 11.24, *p* = 0.001, $\eta_{p}^{2}$ = 0.06]. Male participants also had a higher body mass index than female participants [*F*(1,174) = 41.32, *p* = 0.001, $\eta_{p}^{2}$ = 0.19] but did not differ from female participants on any resting state vmHRV measure [RMSSD-300: *F*(1,174) = 1.40, *p* = 0.249, $\eta_{p}^{2}$ = 0.01; RMSSD-120: *F*(1,173) = 1.78, *p* = 0.184, $\eta_{p}^{2}$ = 0.01; RMSSD-060: *F*(1,173) = 2.03, *p* = 0.156, $\eta_{p}^{2}$ = 0.01]. Female participants reported more psychopathology [*F*(1,174) = 7.87, *p* = 0.006, $\eta_{p}^{2}$ = 0.10] and less reappraisal use than male participants [*F*(1,174) = 19.28, *p* = 0.001, $\eta_{p}^{2}$ = 0.10]. Reports of suppression use, on the contrary, did not differ between male and female participants [*F*(1,174) = 2.20, *p* = 0.140, $\eta_{p}^{2}$ = 0.01].

### Associations Between Short-Term Measures of Heart Rate Variability and Self-Report Measures of Reappraisal or Suppression Use

**FIGURE 1:**
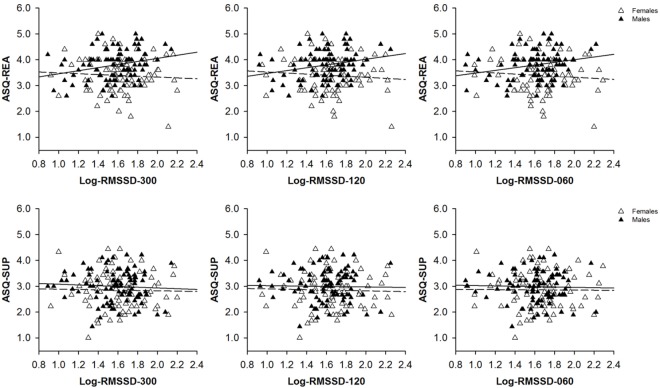
Scatter plots with lines of best fit demonstrating bivariate correlations between self-reported reappraisal (ASQ-REA) or suppression (ASQ-SUP) use and (log-transformed) vagally mediated heartrate variability (Log-RMSSD) that was derived from 300 s (Log-RMSSD-300), 120 s (Log-RMSSD-120), or 60 s (Log-RMSSD-060) lasting resting state recordings of male (black triangulars, solid line) and female (white triangulars, dashed line) participants’ heart rate.

Using resting state vmHRV-ST measures, a series of correlation analyses was run to explore sex-dependent associations between inter-individual differences in resting state vmHRV-ST and inter-individual differences in self-reported emotion regulation abilities. In male participants, inter-individual differences in resting state vmHRV-ST correlated significantly with inter-individual differences in self-reported reappraisal [RMSSD-300: *r*(86) = 0.25, 95% CI [0.05,0.44], *p* = 0.019] but not suppression [RMSSD-300: *r*(86) = -0.08, 95% CI [-0.30,0.14], *p* = 0.476] use. In female participants, there were no significant correlations between inter-individual differences in resting state vmHRV-ST and inter-individual differences in self-reported reappraisal [RMSSD-300: *r*(80) = -0.11, 95% CI [-0.32,0.11], *p* = 0.337] or suppression [RMSSD-300: *r*(80) = 0.02, 95% CI [-0.19,0.21], *p* = 0.858] use. These findings suggested that inter-individual differences in resting state vmHRV-ST may have been differentially associated with inter-individual differences in self-reported emotion regulation abilities in male and female participants (see Figure 1).

To test whether inter-individual differences in resting state vmHRV-ST were in fact differentially associated with inter-individual differences in self-reported emotion regulation abilities in male and female participants, a series of regression analyses was run (see Table 2). A regression analysis with self-reported reappraisal use as the criterion revealed a significant interaction between the predictor sex and the predictor resting state vmHRV-ST [RMSSD-300: *t*(169) = 2.45, *p* = 0.015], a significant effect of the predictor psychopathology [RMSSD-300: *t*(169) = -3.61, *p* = 0.001] and a significant effect of the predictor sex [RMSSD-300: *t*(169) = 3.28, *p* = 0.001]. The effects of the remaining predictors, on the contrary, were all insignificant [RMSSD-300: all *t*(169) ≤|-1.14|, all *p* ≥ 0.343]. A simple slope analysis indicated that resting state vmHRV-ST was a significant predictor of self-reported reappraisal use among male [RMSSD-300: *B* = 0.14, *SE B* = 0.06, 95% CI [0.03,0.26], *t*(86) = 2.39, *p* = 0.017] but not female [RMSSD-300: *B* = -0.07, *SE B* = 0.07, 95% CI [-0.23,0.06], *t*(80) = -0.97, *p* = 0.356] participants. A regression analysis with self-reported suppression use as the criterion failed to reveal a significant interaction between the predictor sex and the predictor resting state vmHRV-ST [RMSSD-300: *t*(169) = -0.32, *p* = 0.751]. The effect of the other predictors, with the exception of the predictor sex [RMSSD-300: *t*(169) = 2.20, *p* = 0.029] and the predictor psychopathology [RMSSD-300: *t*(169) = 4.13, *p* = 0.001], were all insignificant [RMSSD-300: all *t*(169) ≤|-1.42|, all *p* ≥ 0.159]. Taken together, these findings confirmed that there were sex-dependent associations between inter-individual differences in resting state vmHRV-ST and inter-individual differences in self-reported emotion regulation abilities.

**Table 2:** Associations between short-term measures of heartrate variability that had been derived from 300 s lasting heartrate recordings and measures of reappraisal and suppression.

	ASQ-REA					ASQ-SUP				
		
	*B*	*SE B*	95 % CI	*t*	*p*	*B*	*SE B*	95 % CI	*t*	*p*
Step 1										
Sex	0.36	0.11	[0.15, 0.56]	3.42	0.003**	0.24	0.11	[0.05, 0.47]	2.19*	0.030**
Age	-0.02	0.04	[-0.11, 0.06]	-0.47	0.613	-0.07	0.05	[-0.17, 0.03]	-1.44	0.145
BMI	0.00	0.06	[-0.10, 0.12]	0.02	0.982	0.03	0.06	[-0.10, 0.13]	0.56	0.584
BSI-18	-0.16	0.05	[-0.27, -0.06]	-3.39	0.002**	0.21	0.06	[0.07, 0.31]	4.12	0.002**
Log-RMSSD-300	0.04	0.05	[-0.06, 0.13]	0.80	0.475	-0.01	0.05	[-0.11, 0.09]	-0.25	0.824
Step 2										
Sex	0.34	0.11	[0.13, 0.54]	3.28	0.003**	0.25	0.11	[0.05, 0.48]	2.20	0.027*
Age	-0.03	0.04	[-0.11, 0.06]	-0.64	0.503	-0.07	0.05	[-0.17, 0.03]	-1.42	0.160
BMI	0.02	0.06	[-0.09, 0.15]	0.46	0.673	0.03	0.06	[-0.10, 0.14]	0.49	0.633
BSI-18	-0.17	0.05	[-0.28, -0.06]	-3.61	0.003**	0.21	0.06	[0.07, 0.31]	4.13	0.003*
Log-RMSSD-300	-0.07	0.08	[-0.23, 0.07]	-1.14	0.343	0.00	0.07	[-0.14, 0.14]	0.05	0.949
Log-RMSSD-300 × Sex	0.22	0.10	[0.03, 0.42]	2.45	0.019*	-0.03	0.10	[-0.24, 0.17]	-0.32	0.752


*ASQ-REA: *R*^2^ = 0.16, *F*(5,170) = 6.63, *p* = 0.001, Δ*R*^2^ = 0.03, Δ*F*(6,169) = 6.01, *p* = 0.015, ASQ-SUP: *R*^2^ = 0.13, *F*(5,170) = 4.87, *p* = 0.001, Δ*R*^2^ = 0.00, Δ*F*(6,169) = 0.10, *p* = 0.751. ASQ-REA, Affective Style Questionnaire – Reappraisal Scale [42, 43]; ASQ-SUP, Affective Style Questionnaire – Suppression Scale [42, 43]; BMI, body mass index; BSI-18, Brief Symptom Inventory 18 [41]; 95% CI, 95% confidence interval; Log-RMSSD-300, log-transformed root mean square of successive differences between consecutive heart beats that had been derived from 300 s lasting heartrate recordings. ^∗^*p* ≤ 0.05, ^∗∗^*p* ≤ 0.01, ^∗∗∗^*p* ≤ 0.001.*

### Associations Between Ultra-Short-Term Measures of Heart Rate Variability and Self-Report Measures of Reappraisal or Suppression Use

Using resting state vmHRV-UT measures, another series of correlation analyses was run to explore sex-dependent associations between inter-individual differences in resting state vmHRV-UT and inter-individual differences in self-reported emotion regulation abilities. These analyses yielded similar findings like those that have been obtained in the correlation analyses that used resting state vmHRV-ST measures. In male participants, inter-individual differences in resting state vmHRV-UT were significantly correlated with inter-individual differences in self-reported reappraisal [RMSSD-120: *r*(85) = 0.23, 95% CI [0.03,0.44], *p* = 0.031; RMSSD-060: *r*(85) = 0.21, 95% CI [0.02,0.39], *p* = 0.05] but not suppression [RMSSD-120: *r*(85) = -0.04, 95% CI [-0.24,0.18], *p* = 0.702; RMSSD-060: *r*(85) = -0.05, 95% CI [-0.25,0.18], *p* = 0.679] use. In female participants, inter-individual differences in resting state vmHRV-UT correlated neither with inter-individual differences in self-reported reappraisal use [RMSSD-120: *r*(80) = -0.12, 95% CI [-0.33,0.10], *p* = 0.272; RMSSD-060: *r*(80) = -0.18, 95% CI [-0.31,0.09], *p* = 0.297] nor with inter-individual differences in self-reported suppression use [RMSSD-120: *r*(80) = 0.02, 95% CI [-0.20,0.21], *p* = 0.884; RMSSD-060: *r*(86) = 0.02, 95% CI [-0.18,0.22], *p* = 0.836]. According to these findings, inter-individual differences in resting state vmHRV-UT may have been differentially associated with inter-individual differences in self-reported emotion regulation abilities in male and female participants (see Figure 1).

A series of regression analyses was run to test whether inter-individual differences in resting state vmHRV-UST were in fact differentially associated with inter-individual differences in self-reported emotion regulation abilities (see Tables 3, 4). These analyses revealed similar findings like those that have been found in the regression analyses that used resting state vmHRV-ST measures. A regression analysis with self-reported reappraisal use as the criterion revealed a significant interaction between the predictor sex and the predictor resting state vmHRV-UT [RMSSD-120: *t*(168) = 2.45, *p* = 0.015, RMSSD-060: *t*(168) = 2.30, *p* = 0.023], a significant effect of the predictor psychopathology [RMSSD-120: *t*(168) = -3.67, *p* = 0.001, RMSSD-060: *t*(168) = -3.61, *p* = 0.001] and a significant effect of the predictor sex [RMSSD-120: *t*(168) = 3.29, *p* = 0.001, RMSSD-060: *t*(168) = 3.25, *p* = 0.001]. The effects of the other predictors were all insignificant [RMSSD-120: all *t*(168) ≤|-1.32|, all *p* ≥ 0.189, RMSSD-060: all *t*(168) ≤|-1.23|, all *p* ≥ 0.221]. A simple slope analysis showed that resting state vmHRV-UT was a significant predictor of self-reported reappraisal use in male [RMSSD-120: *B* = 0.13, *SE B* = 0.06, 95% CI [0.02,0.25], *t*(85) = 2.20, *p* = 0.026; RMSSD-060: *B* = 0.12, *SE B* = 0.06, 95% CI [0.01,0.24], *t*(85) = 1.99, *p* = 0.035] but not female [RMSSD-120: *B* = -0.08, *SE B* = 0.07, 95% CI [-0.23,0.07], *t*(80) = -1.11, *p* = 0.294; RMSSD-060: *B* = -0.07, *SE B* = 0.07, 95% CI [-0.21, 0.05], *t*(80) = -1.05, *p* = 0.276] participants. A regression analysis with suppression use as the criterion found no significant interaction between the predictor sex and the predictor resting state vmHRV-UT [RMSSD-120: *t*(168) = -0.01, *p* = 0.926, RMSSD-060: *t*(168) = -0.11, *p* = 0.912] but a significant effect of the predictor sex [RMSSD-120: *t*(168) = 2.18, *p* = 0.031, RMSSD-060: *t*(168) = 2.19, *p* = 0.030] and a significant effect of the predictor psychopathology [RMSSD-120: *t*(168) = 4.10, *p* = 0.001, RMSSD-060: *t*(168) = 4.12, *p* = 0.001]. The effects of the remaining predictors were not significant [RMSSD-120: all *t*(168) ≤|-1.42|, all *p* ≥ 0.158; RMSSD-060: all *t*(168) ≤|-1.40|, all *p* ≥ 0.162]. Taken together, these findings confirmed that there were sex-dependent associations between inter-individual differences in resting state vmHRV-UT and inter-individual differences in emotion regulation abilities.

**Table 3:** Associations between short-term measures of heartrate variability that had been derived from 120 s lasting heartrate recordings and measures of reappraisal and suppression.

	ASQ-REA					ASQ-SUP				
		
	*B*	*SE B*	95% CI	*t*	*p*	*B*	*SE B*	95% CI	*t*	
Step 1										
Sex	0.35	0.11	[0.13, 0.55]	3.36	0.002**	0.24	0.11	[0.04, 0.44]	2.18	0.029*
Age	-0.02	0.05	[-0.11, 0.07]	-0.50	0.595	-0.07	0.05	[-0.17, 0.03]	-1.43	0.151
BMI	0.00	0.06	[-0.11, 0.13]	-0.04	0.969	0.03	0.06	[-0.09, 0.14]	0.57	0.575
BSI-18	-0.16	0.05	[-0.27, -0.06]	-3.47	0.003**	0.21	0.06	[0.08, 0.31]	4.12	0.001**
Log-RMSSD-120^a^	0.02	0.05	[-0.08, 0.12]	0.48	0.652	-0.01	0.05	[-0.10, 0.09]	-0.13	0.884
Step 2										
Sex	0.34	0.11	[0.13, 0.55]	3.29	0.002**	0.24	0.11	[0.04, 0.45]	2.18	0.028*
Age	-0.03	0.05	[-0.12, 0.07]	-0.61	0.537	-0.07	0.05	[-0.17, 0.03]	-1.42	0.155
BMI	0.01	0.06	[-0.10, 0.14]	0.25	0.839	0.03	0.06	[-0.09, 0.14]	0.55	0.586
BSI-18	-0.17	0.05	[-0.27, -0.06]	-3.67	0.002**	0.21	0.06	[0.08, 0.31]	4.10	0.001**
Log-RMSSD-120^a^	-0.08	0.08	[-0.23, 0.06]	-1.32	0.276	0.00	0.07	[-0.14, 0.14]	-0.03	0.981
Log-RMSSD-120^a^ × Sex	0.22	0.09	[0.05, 0.41]	2.45	0.015*	-0.01	0.10	[-0.19, 0.18]	-0.09	0.932


*ASQ-REA: *R*^2^ = 0.16, *F*(5,169) = 6.50, *p* = 0.001, Δ*R*^2^ = 0.03, Δ*F*(6,168) = 6.02, *p* = 0.015, ASQ-SUP: *R*^2^ = 0.12, *F*(5,169) = 4.79, *p* = 0.001, Δ*R*^2^ = 0.00, Δ*F*(6,168) = 0.01, *p* = 0.926. ASQ-REA, Affective Style Questionnaire – Reappraisal Scale [42, 43]; ASQ-SUP, Affective Style Questionnaire – Suppression Scale [42, 43]; BMI, body mass index; BSI-18, Brief Symptom Inventory 18 [41]; 95% CI, 95% confidence interval; Log-RMSSD-120, log transformed root mean square of successive differences between consecutive heart beats that had been derived from 120 s lasting heartrate recordings. ^*a*^Data was missing for one male participant due to technical difficulties. ^∗^*p* ≤ 0.05, ^∗∗^*p* ≤ 0.01, ^∗∗∗^*p* ≤ 0.001.*

**Table 4:** Associations between ultra-short term measures of heartrate variability that had been derived from 60 s lasting heartrate recordings and measures of reappraisal and suppression use.

	ASQ-REA					ASQ-SUP				
		
	*B*	*SE B*	95% CI	*t*	*p*	*B*	*SE B*	95% CI	*t*	
Step 1										
Sex	0.35	0.10	[0.15, 0.54]	0.3.36	0.003**	0.24	0.11	[0.02, 0.46]	2.19	0.040*
Age	-0.02	0.05	[-0.11, 0.07]	-0.50	0.627	-0.07	0.05	[-0.17, 0.03]	-1.41	0.162
BMI	0.00	0.06	[-0.11, 0.13]	-0.02	0.991	0.03	0.06	[-0.09, 0.14]	0.58	0.577
BSI-18	-0.16	0.05	[-0.27, -0.08]	-3.46	0.003**	0.21	0.06	[0.08, 0.31]	4.13	0.002
Log-RMSSD-60	0.02	0.05	[-0.07, 0.12]	0.50	0.637	0.00	0.05	[-0.10, 0.10]	-0.01	0.994
Step 2										
Sex	0.34	0.10	[0.13, 0.53]	3.25	0.003	0.24	0.11	[0.02, 0.47]	2.19	0.041*
Age	-0.03	0.05	[-0.12, 0.07]	-0.52	0.612	-0.07	0.05	[-0.17, 0.03]	-1.40	0.158
BMI	0.02	0.06	[-0.10, 0.15]	0.30	0.791	0.03	0.06	[-0.09, 0.15]	0.55	0.595
BSI-18	-0.17	0.05	[-0.28, -0.08]	-3.61	0.002**	0.21	0.06	[0.08, 0.32]	4.12	0.002**
Log-RMSSD-60^a^	-0.08	0.07	[-0.22, 0.06]	-1.23	0.242	0.01	0.07	[-0.14, 0.13]	0.07	0.947
Log-RMSSD-60^a^ × Sex	0.21	0.09	[0.04, 0.39]	2.30	0.016*	-0.01	0.09	[-0.20, 0.18]	-0.11	0.898


*ASQ-REA: *R*^2^ = 0.16, *F*(5,169) = 6.50, *p* = 0.001, Δ*R*^2^ = 0.03, Δ*F*(6,168) = 5.30, *p* = 0.023, ASQ-SUP: *R*^2^ = 0.12, *F*(5,169) = 4.79, *p* = 0.001, Δ*R*^2^ = 0.00, Δ*F*(6,168) = 0.01, *p* = 0.912. ASQ-REA, Affective Style Questionnaire – Reappraisal Scale [42, 43]; ASQ-SUP, Affective Style Questionnaire – Suppression Scale [42, 43]; BMI, body mass index; BSI-18, Brief Symptom Inventory 18 [41]; 95% CI, 95% confidence interval; Log-RMSSD-60, log transformed root mean square of successive differences between consecutive heart beats that had been derived from 60 s lasting heartrate recordings. ^a^Data was missing for one male participant due to technical difficulties. ^∗^*p* ≤ 0.05, ^∗∗^*p* ≤ 0.01, ^∗∗∗^*p* ≤ 0.001.*

### Correspondence Between (Ultra-)Short-Term Measures of Heart Rate Variability

**FIGURE 2:**
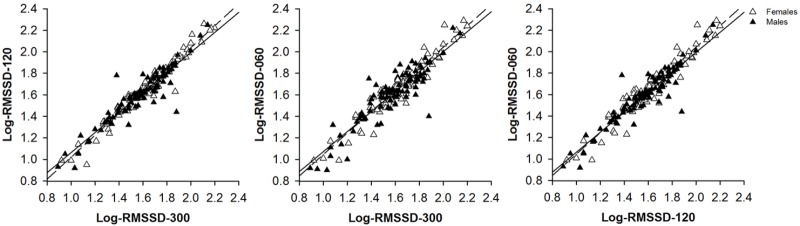
Scatter plots with lines of best fit demonstrating bivariate correlations between (log-transformed) vagally mediated heartrate variability (Log-RMSSD) that was derived from 300 s (Log-RMSSD-300), 120 s (Log-RMSSD-120), or 60 s (Log-RMSSD-060) lasting resting state recordings of male (black triangulars, solid line) and female (white triangulars, dashed line) participants’ heart rate.

Bivariate correlations and intra-class correlations were used to analyze the correspondence between resting state vmHRV-ST and vmHRV-UT measures (see Table 5 and Figure 2). The respective correlation coefficients indicated a high correspondence between the different resting state vmHRV measures, in male [all *r* ≥ 0.90, all ICC ≥ 0.95] as well as in female [all *r* ≥ 0.96, all ICC ≥ 0.97] participants.

**Table 5:** Correspondence between short-term and ultra-short-term measures of heart rate variability.

	Female participants (*n* = 85)				Male participants (*n* = 90)			
	***r***	**95% CI**	**ICC**	**95% CI**	***r***	**95% CI**	**ICC**	**95% CI**
Log-RMSSD-300 vs. Log-RMSSD-120^a^	0.98	[0.96,0.99]	0.98	[0.96,0.99]	0.94	[0.87,0.97]	0.96	[0.94,0.98]
Log-RMSSD-300 vs. Log-RMSSD-060^a^	0.96	[0.94,0.97]	0.97	[0.93,0.99]	0.90	[0.83,0.95]	0.95	[0.91,0.97]
Log-RMSSD-120^a^ vs. Log-RMSSD-060^a^	0.98	[0.96,0.99]	0.99	[0.98,0.99]	0.97	[0.95,0.98]	0.98	[0.98,0.99]


*95% CI, 95% confidence interval; Log-RMSSD-300, root mean square of successive differences between consecutive heart beats that had been derived from 300 s lasting heartrate recordings; Log-RMSSD-120, root mean square of successive differences between consecutive heart beats that had been derived from 120 s lasting heartrate recordings; Log-RMSSD-60, root mean square of successive differences between consecutive heart beats that had been derived from 60 s lasting heartrate recordings. ^*a*^Data was missing for one male participant due to technical difficulties.*

## Discussion

In the present study, we investigated whether inter-individual differences in resting state vmHRV would be associated with inter-individual differences in self-reported emotion regulation abilities. Across a series of correlation and regression analyses, we found sex-dependent associations between inter-individual differences in resting state vmHRV and inter-individual differences in self-reported reappraisal use but not suppression use: Male participants with high resting state vmHRV reported more reappraisal but similar suppression use than male participants with low resting state vmHRV, indicating an increase in self-reported reappraisal but not suppression use with increasing resting state vmHRV. Female participants with high and low resting state vmHRV, on the contrary, did not differ in self-reported reappraisal or suppression use, implying that self-reported reappraisal and suppression use did not increase or decrease with increasing or decreasing resting state vmHRV. Of note, the aforementioned associations between inter-individual differences in resting state vmHRV and inter-individual differences in self-reported emotion regulation abilities emerged not only in correlation but also in regression analyses that involved short-term as well as ultra-short term measures of resting state vmHRV, indicating the robustness of our findings.

Our findings regarding sex-differences in self-reported emotion regulation abilities are consistent with those of previous studies revealing more self-reported reappraisal and suppression use in male as compared to female participants (Graser et al., 2012; Erreygers and Spooren, 2017; Totzeck et al., 2018). Moreover, our findings complement findings of other studies indicating that female participants report and show more emotionality than male participants (Grossman and Wood, 1993; Kring and Gordon, 1998; Bradley et al., 2001), implying that sex-differences in emotion regulation may account for sex-differences in emotional sensitivity and emotional expressivity. Notwithstanding the role of sex-differences in emotion regulation, it is interesting to note that our findings converge with the findings of a study that investigated the association of inter-individual differences in HRV with inter-individual differences in self-reports regarding the *inability* rather than *ability* to regulate emotions (Williams et al., 2015). In that study, participants with high vmHRV reported fewer difficulties to understand and control emotions than participants with low vmHRV (Williams et al., 2015). As an understanding and control of emotions is more relevant for reappraisal than suppression use (Hofmann and Kashdan, 2010; Totzeck et al., 2018), the findings of that study indicate a similar association of inter-individual differences in vmHRV with inter-individual differences in emotion regulation like the one that has been found in the present study. Although the findings of these studies suggest that inter-individual differences in vmHRV are more associated with inter-individual differences in self-reported reappraisal than self-reported suppression use, it is important to note that both studies relied on self-report measures that lack ecologic validity in comparison to performance measures. Studies that used performance measures, however, revealed similar associations of inter-individual differences in vmHRV with inter-individual differences in reappraisal use (Butler et al., 2006; Vogele et al., 2010; Volokhov and Demaree, 2010; Denson et al., 2012; Berna et al., 2014; Williams et al., 2015). Moreover, these associations were not only found in studies that measured inter-individual differences in vmHRV during rest (Vogele et al., 2010; Volokhov and Demaree, 2010; Williams et al., 2015) but also in studies that measured inter-individual differences in vmHRV during performance (Butler et al., 2006; Denson et al., 2012; Berna et al., 2014). Methodological aspects regarding the measurement of inter-individual differences in HRV and emotion regulation, thus, do not affect the association between inter-individual differences in vmHRV and inter-individual differences in reappraisal use, implying that this association is remarkably robust. Inter-individual differences in vmHRV may, therefore, indeed have the potential to work as a biomarker for inter-individual differences in emotion regulation (Appelhans and Luecken, 2006; Beauchaine, 2015; Holzman and Bridgett, 2017).

With respect to the neurobiological mechanisms underlying associations between inter-individual differences in resting state vmHRV and inter-individual differences in emotion regulation, it is interesting to note that inter-individual differences regarding the activity and integrity of prefrontal and (para-)limbic brain regions are associated with inter-individual differences in emotion regulation (Ochsner et al., 2012; Frank et al., 2014; Etkin et al., 2015) as well as with inter-individual differences in vmHRV (Thayer and Lane, 2009; Thayer et al., 2012; Smith et al., 2017). Of these brain regions, prefrontal ones, like, for example, the dorsolateral and ventrolateral prefrontal cortex or the anterior cingulate cortex, and (para-)limbic ones, like, for example, the amygdala and the insula, appear to be of particular relevance. Imaging studies revealed that an increase in prefrontal activity and a decrease in (para-)limbic activity is more likely to occur during reappraisal than suppression use (Goldin et al., 2008; Drabant et al., 2009; McRae et al., 2010; Vanderhasselt et al., 2013; Nelson et al., 2015), indicating a more efficient inhibition of (para-)limbic brain regions by prefrontal brain regions via an increased coupling of these brain regions in the context of reappraisal use (Ochsner et al., 2002; Banks et al., 2007; Morawetz et al., 2017). However, imaging studies also revealed that an increase in prefrontal activity and a decrease in (para-)limbic activity is accompanied by an increase in vmHRV (Gianaros et al., 2004; Lane et al., 2009; Sakaki et al., 2016). These studies even showed that an increased coupling of prefrontal and (para-)limbic brain regions, which is thought to be crucial for successful emotion regulation following reappraisal use (Ochsner et al., 2012), is also accompanied by an increase in vmHRV (Sakaki et al., 2016). On basis of these findings, it may be assumed that inter-individual differences in vmHRV reflect inter-individual differences in reappraisal use that are mediated by inter-individual differences regarding the inhibition of (para-)limbic brain regions by prefrontal brain regions. Inter-individual differences in vmHRV may, thus, indeed serve as a biomarker for inter-individual differences in emotion regulation (Appelhans and Luecken, 2006; Beauchaine, 2015; Holzman and Bridgett, 2017).

Despite the plausibility of these assumptions, it has to be acknowledged that they are only partially supported by the findings of the present and previous studies. Although previous studies demonstrated that inter-individual differences in prefrontal-(para-)limbic engagement are associated with inter-individual differences vmHRV and that inter-individual differences in prefrontal-(para-)limbic engagement are associated with inter-individual differences in emotion regulation, these studies *either* focused on inter-individual differences in emotion regulation (Goldin et al., 2008; Drabant et al., 2009; McRae et al., 2010; Vanderhasselt et al., 2013; Nelson et al., 2015) *or* on inter-individual differences in vmHRV (Gianaros et al., 2004; Lane et al., 2009; Sakaki et al., 2016). It, thus, remains open whether inter-individual differences in prefrontal-(para-)limbic engagement in fact account for associations between inter-individual differences in vmHRV and inter-individual differences in emotion regulation. It is also unclear whether the assumed associations emerge in a similar way under active as under passive conditions because previous studies *either* employed resting state (Williams et al., 2015) *or* task based (Butler et al., 2006; Vogele et al., 2010; Volokhov and Demaree, 2010; Denson et al., 2012; Berna et al., 2014) measures in their investigations. Consequently, there is a need for a *combined* assessment of inter-individual differences in prefrontal-(para-)limbic engagement, inter-individual differences in vmHRV and inter-individual differences in emotion regulation under various conditions, passive as well as active ones. In this respect, it is noteworthy that previous studies measured inter-individual differences in vmHRV on basis of HRV measures that were derived from short-term HR recordings (Butler et al., 2006; Vogele et al., 2010; Volokhov and Demaree, 2010; Denson et al., 2012; Berna et al., 2014; Williams et al., 2015) and that the present study measured inter-individual differences in vmHRV on basis of HRV measures that were derived from short-term as well as ultra-short-term HR recordings. Although the findings of the present and previous studies suggest a high correspondence between short-term and ultra-short-term vmHRV measures (Munoz et al., 2015; Lischke et al., 2018b), it remains to be determined whether short-term measures can be substituted by ultra-short-term measures in studies like the present one (Pecchia et al., 2018). Future studies that assess the correspondence between ultra-short-term and short-term vmHRV measures in larger participant samples with more sophisticated methods than in the present study may be useful to delineate the conditions under which ultra-short-term vmHRV measures can be used as a substitute for short-term vmHRV measures. However, vmHRV measures may not be the only measure that may be useful for an assessment of inter-individual differences in emotion regulation. Previous studies revealed associations between inter-individual differences in pupil size (PLS) and inter-individual differences in emotion regulation that were mediated by inter-individual differences in prefrontal-(para-)limbic engagement (Urry et al., 2006, 2009), implying that inter-individual differences in pupil size may also work as a biomarker for inter-individual differences in emotion regulation. Moreover, previous studies also suggest that inter-individual differences in PLS co-vary with inter-individual differences in vmHRV (Park et al., 2018). It may, thus, be worthwhile to consider not only measures of vmHRV but also measures of PLS in future studies that are concerned with the identification of brain-based biomarkers of emotion regulation.

Notwithstanding that further studies are needed to replicate and extend the findings of the present study, we tentatively suggest that the neurobiological mechanisms underlying inter-individual differences in emotion regulation do not necessarily have to be investigated with techniques that require dedicated staff or equipment (Beauchaine, 2015). First of all, inter-individual differences in prefrontal-(para-)limbic control that account for inter-individual differences in emotion regulation may be assessed with measurements of cardiac activity that are less time- and resource-consuming than measurements of neural activity. Second, measurements of cardiac activity that represent inter-individual differences in vmHRV may be assessed on basis of ultra-short-term resting state vmHR recordings. There was not only a remarkable correspondence between ultra-short-term and short-term measurements of resting state vmHRV regarding the measurement of inter-individual differences in resting state vmHRV but also regarding the association between inter-individual differences in resting state vmHRV and inter-individual differences in self-reported emotion regulation abilities. Ultra-short-term measures of resting state vmHRV have already been shown to be a valid a reliable alternative to short-term measures of resting state vmHRV (Munoz et al., 2015; Lischke et al., 2018b), indicating that ultra-short term measures may be used as time-saving alternative to short-term measures under certain conditions. Third, inter-individual differences in emotion regulation may be assessed on basis of self-report measures whose scores are associated with inter-individual differences in emotion regulation on the behavioral and neurobiological level (Drabant et al., 2009; Szasz et al., 2011; Nelson et al., 2015). Well-validated self-report measures, such as the ASQ (Hofmann and Kashdan, 2010; Graser et al., 2012) or ERQ (Gross and John, 2003; Abler and Kessler, 2009), may be used as a time- and resource-saving alternative to more complex emotion regulation tasks (Gross, 1998; Hofmann et al., 2009). Combining (ultra-)short-term measures of resting state vmHRV with short self-report measures of emotion regulation may, therefore, be interesting for researchers who need to investigate the neurobiological mechanisms underlying inter-individual differences in emotion regulation in a time- and resource-efficient manner. However, even if time and resources are not scarce, it may be valuable to combine the aforementioned measures with other measures to fully elucidate the behavioral and neurobiological correlates of emotion regulation.

In the present study, we found sex-specific associations between inter-individual differences in resting state vmHRV and inter-individual differences in self-reported reappraisal but not suppression use. As we did not assess neural and behavioral correlates of emotion regulation in our study, we can only assume that these associations reflect inter-individual differences in prefrontal-(para-)limbic engagement during emotion regulation (Thayer and Lane, 2009; Thayer et al., 2012; Smith et al., 2017). However, similar associations between inter-individual differences in emotion regulation and inter-individual differences in vmHRV have been found in previous studies (Butler et al., 2006; Denson et al., 2012; Vogele et al., 2010; Volokhov and Demaree, 2010; Berna et al., 2014; Williams et al., 2015), supporting theoretical claims that inter-individual differences in vmHRV serve as a biomarker for inter-individual differences in emotion regulation (Appelhans and Luecken, 2006; Beauchaine, 2015; Holzman and Bridgett, 2017). Future studies are now warranted that further investigate whether measurements of cardiac activity are in fact a time- and resource-saving alternative to measurements of neural activity in the search for biomarkers indicating deficits in emotion regulation (Beauchaine, 2015).

## Author Contributions

AL and RP designed the study. AL, AM-M, MW, RJ, and SP collected the data. AL and RP analyzed the data. AL and RP wrote the manuscript. AH, AM-M, MW, RJ, and SP contributed to writing, reviewing and editing of the manuscript. All authors approved the final version of the manuscript.

## Conflict of Interest Statement

The authors declare that the research was conducted in the absence of any commercial or financial relationships that could be construed as a potential conflict of interest.
